# Injury incidence, determinants, and associated burden among children aged 0–17 years: a cross-sectional study in Henan Province, Central China

**DOI:** 10.3389/fpubh.2026.1794442

**Published:** 2026-03-10

**Authors:** Bingyuan Wang, Wenjie Yang, Luwei Qin, Pan Pan, Tianfang Xing, Shaofang Li, Xiujuan Di, Zhiwei Han, Linqi Diao

**Affiliations:** Henan Provincial Center for Disease Control and Prevention, Zhengzhou, Henan, China

**Keywords:** burden, childhood injury, China, cross-sectional study, epidemiology, risk factors

## Abstract

**Background:**

Childhood injury is a major public health issue in China, yet detailed provincial-level data are limited. This study aimed to investigate the epidemiology, determinants, and burden of injuries among children aged 0–17 years in Henan Province.

**Methods:**

A population-based cross-sectional survey was conducted from June 2022 to May 2023 using a multi-stage stratified cluster random sampling design. Data were collected via face-to-face questionnaires from guardians of 9,859 children aged 0–17 years. Complex sampling weights were applied, and Rao-Scott *χ*^2^ tests and logistic regression were used for analysis.

**Results:**

The overall injury incidence rate was 6.88%, with a significantly higher rate in boys (8.23%) than in girls (5.32%). Multivariable logistic regression indicated that boys had higher odds of injury compared to girls (OR = 1.58, 95% CI: 1.27–1.97), and day students (returning home daily) had higher odds compared to children not enrolled in school (OR = 1.69, 95% CI: 1.16–2.46). Children under the care of their grandparents (OR = 1.33, 95%CI: 1.06–1.67) had a higher injury risk compared to those cared for by their mother. Critically, although a caregiver was present at the time of injury in 54.17% of incidents, effective supervision was lacking in 67.71% of these cases. Falls were the leading cause (53.90% of all incidents) of injury. The home was the most common injury location (41.73%), and leisure activities were the most frequent circumstance (59.30%). The most common injury type was contusion/abrasion (37.47%); the lower extremities were the most frequently injured body site (37.70%). The mean hospital stay was 18 days, with an average of 9 missed days, and the mean cost per injury was 2,713 Chinese Yuan, with road traffic injuries incurring the highest costs.

**Conclusion:**

This study provides key evidence for childhood injury prevention in Henan Province. Priorities include strengthening supervision (especially at home during leisure), tailored interventions for high-risk groups (boys, day students, grandparent-care children), and focusing on falls (high frequency) and road traffic injuries (high cost). Implementing these strategies is essential to reduce the burden of preventable childhood injuries.

## Introduction

Injury remains a leading global cause of death and a major contributor to morbidity, long-term disability, and healthcare costs in children and adolescents (1–3). During the period from 2010 to 2021, injuries caused more than 4.4 million deaths worldwide on average each year, including over 300,000 deaths annually among children aged 0–14 years (4). Globally, economic losses attributable to injuries from 2015 to 2030 are estimated at approximately US$7.86 trillion. Low- and middle-income countries (LMICs) are anticipated to bear a disproportionate share of this burden, with projected losses nearly 50% greater than those in high-income countries (HICs) (5). As a developing country, China also faces significant challenges in childhood injury prevention. Since 1990, although the mortality rate and disability-adjusted life years (DALYs) due to childhood injuries in China have shown a declining trend, the overall incidence of childhood injuries has been increasing, and injury remains the primary cause of death for children aged 1–14 years (6–8). This suggested that although injury related mortality has declined, the occurrence itself remains a widespread challenge.

In response to this challenge and to safeguard healthy child development, China has prioritized childhood injury prevention at a national level. Key policy documents such as the “Healthy China 2030” blueprint and the “China Children’s Development Program (2021–2030)” explicitly call for reducing injury-related deaths and disabilities and strengthening corresponding prevention and intervention efforts (9, 10). However, translating these national goals into effective local action relies heavily on a thorough understanding of the specific situation on the ground.

Nevertheless, the research on the incidence, characteristics, and associated socioeconomic burden of childhood injuries was limited at the provincial level in China, particularly in Henan, which has a large population. This information gap hinders the development of precise and effective prevention strategies. Therefore, to provide a scientific basis for childhood injury prevention and control in Henan Province, this study utilizes data from the 2022–2023 Henan provincial resident injury survey. It aims to systematically describe and analyze the epidemiological characteristics, clinical profiles, and the multifaceted burden of injuries among children aged 0–17 years in the province, thereby offering evidence to inform targeted interventions.

## Materials and methods

### Study design and populations

This study was a population-based, cross-sectional study conducted in Henan Province, a central region of China. Participants were recruited using a multi-stage stratified cluster random sampling method from June to October 2023, and were asked to retrospectively report injury events that occurred the 12 months period from during June 1 of 2022 and May 31 of 2023. First, all counties (districts) in Henan Province were stratified into 8 strata based on urbanization rate (high/low), population size (high/low), and mortality rate (high/low), and 14 disease surveillance points (DSPs) were selected using stratified sampling. Second, with each DSP, 3 townships (streets/regimental units) were selected using probability proportional to size (PPS) systematic sampling. Third, from each selected township, 2 villages were selected using PPS systematic sampling. Fourth, within each selected village, one resident group (comprising at least 150 households) was chosen by simple random sampling. Finally, from each resident group, 125 household units were randomly selected using simple random sampling.

Based on this design, the minimum required sample size was 750 households per DSP (3 townships × 2 villages × 125 households), resulting in a provincial minimum of 10,500 households (14 DSPs × 750 households). To ensure representativeness, post-sampling adjustments were applied based on the gender ratio from the Seventh National Population Census of Henan Province. If a DSP failed to meet the gender ratio requirement, additional households were sequentially recruited from the remaining households in the corresponding resident group until the requirement was satisfied. Ultimately, we recruited 10,988 households, comprising a total of 39,300 participants with no age restrictions, and the questionnaire response rate was 98.18%, defined as the proportion of eligible sampled households that completed the survey.

Participants were excluded if they refused to answer the questionnaire or sign the informed consent form. The study protocol was reviewed and approved by the Medical Ethics Committee of the Henan Center for Disease Control and Prevention (Approval No.: 2023-KY-003-02). Written informed consent was obtained from all participants prior to the survey. For the present study, we included data from 9,859 children aged 0–17 years, following the definition of a child established by the United Nations Convention on the Rights of the Child (11).

### Sample size calculation

The sample size was calculated using the formula recommended in the Guidelines for Conducing Community Surveys on Injuries and Violence: $N=[4(r)(1-r)(f)(1.1)]/[e^{2}(P)(n)]\;$ (12), where r is the expected injury incidence rate for this survey. This was set at 5.6%, based on the non-fatal injury incidence rate reported among residents of Jiangxi Province (13); *n* is the average number of persons per household. According to the 2020 population census data of Henan Province, the average urban resident household size was 3.24; *f* = 2, *e* = 3%, *p* = 1. Using the above parameter values, the calculated number of households required per stratum was approximately 160. Considering the three stratification factors-urban/rural residence (2 levels), gender (2 levels), and age (16 strata); the total minimum number of households for the survey was determined as: 160 × 2 × 2 × 16 = 10,240 households. Therefore, the sample size of this study was sufficient.

### Baseline examination and data collection

A standardized questionnaire was developed based on the National Injury Surveillance System (NISS) of China (14) and Guidelines for Conducing Community Surveys on Injuries and Violence (12). The questionnaire comprised demographic information, household risk factors, injury occurrence, clinical details of injuries, and the burden of injuries. Trained professionals from disease prevention and control agencies conducted the surveys. Data on injury incidents among residents from June 1, 2022 to May 31, 2023, were collected through face-to-face questionnaire interviews, supplemented by a review of medical records from village clinics, township health centers, and emergency/outpatient departments of local hospitals, as well as health insurance claim information. Within each household, the senior or eldest female member present at the time of the interview was generally designated as the respondent. Whenever possible, the head of the household along with other available members were encouraged to be present to corroborate or elaborate on the provided information. In cases involving a child’s injury event, the respondent was specifically the primary caregiver, most often the mother.

Household members were presented with a comprehensive list of injury types-including falls, road traffic injuries, blunt force injuries, firearm injuries, sharp object injuries, burns or scalds, suffocation, drowning, poisoning, animal-related injuries, and sexual assault-and asked whether any such event had resulted in injury or death during the preceding year.

### Injury definition and classification

Injury was defined according to the World Health Organization (WHO) and the China National Injury Surveillance System (NISS) as physical damage to the human body resulting from acute exposure to energy or a lack of a vital element (12, 14). For the purpose of this population-based survey, an operational case definition was adopted in accordance with the injury definition established by the Injury Prevention and Control Branch of the Chinese Medical Association (15, 16), an injury case was defined as meeting either of the following two criteria: (1) having received a medical diagnosis for the injury at a healthcare facility; or (2) having taken one or more days off from work, school, or usual activities due to the injury.

Injury types were classified according to the NISS framework and included: falls, road traffic injuries, blunt force injuries, sharp object injuries, animal-related injuries, burns/scalds, and other injuries (14). Injury intent was also categorized following NISS standards into four groups: unintentional, self-harm/suicide, intentional, and unclear (14).

### Quality control

A standardized quality control protocol was implemented across all survey sites. The study protocol, manual, and questionnaire were uniformly specified. All investigators received centralized training and certification; eligible investigators held a background in public health or medicine, at least 1 year of epidemiological research experience, and familiarity with local dialects. Data were collected using an electronic questionnaire system with audio recording. Completed questionnaires and audio files were uploaded daily. County/district and municipal CDCs each randomly sampled 20% of electronic questionnaires for audio verification, identified errors were promptly corrected or re-investigated.

### Statistical analysis

This study employed a multi-stage complex sampling design, and all analytical results were adjusted using weighting methods appropriate for complex survey designs. The complex sampling weights comprised the design weight, the non-response weight, and the post-stratification weight (17). The design weight was calculated as the product of the sampling weights from each stage. Upon completion of the survey, the non-response and post-stratification weights were computed based on the actual response rates and the demographic structure of the sample. The non-response weight for a household was defined as the total number of eligible individuals in the household divided by the total number who actually completed the personal questionnaire. The post-stratification weight was calculated by cross-classifying the sample with data from the Seventh National Population Census of Henan Province into strata defined by age group (18 categories), sex (2 categories), and urban–rural residence (2 categories).

Injury occurrence was evaluated using the injury incidence rate and the injury event rate. The injury incidence rate was calculated as the number of injured individuals divided by the total surveyed population, while the injury event rate was calculated as the total number of injury events divided by the total surveyed population. Weighted incidence of injuries in the children and different strata of children was calculated. Differences in proportions between groups were tested using the Rao-Scott chi-squared test for weighted incidence (18, 19). For the weighted incidence estimations and weighted logistic regression analyses, the STRATA, CLUSTER, and WEIGHT statements in survey procedure were used to produce provincial representative estimates. The burden of childhood injuries was assessed using indicators related to medical treatment, hospitalization, days off due to injury, and economic losses (20, 21).

All data cleaning and analyses were performed using SAS 9.4 (SAS Inst., Cary, NC, USA), Two-sided *p* < 0.05 was considered statistically significant.

## Results

### Overall injury incidence and distribution by demographic characteristics

A total of 9,859 children aged 0–17 years were surveyed, with an overall injury incidence rate of 6.88%. The injury incidence rate was significantly higher in boys (8.23%) than in girls (5.32%) (*χ*^2^ = 40.33, *p* < 0.001). Analysis by age group showed no statistically significant difference in injury incidence among children aged 0–4, 5–9, 10–14, and 15–17 years (*p* = 0.873). However, significant differences were observed by education level: kindergarten children had the highest injury incidence rate (8.86%), followed by middle school students (6.91%), high school or above students (6.71%), primary school students (6.55%), and children not enrolled in kindergarten/school who had the lowest rate (4.94%) (*χ*^2^ = 11.76, *p* = 0.019). No statistically significant difference was found between urban and rural children (*p* = 0.924) (Table 1).

**Table 1:** Injury incidence rate by gender, age, residence, and education status among children in Henan Province, China.

Groups	No. of injured individuals	Injury incidence rate (%, weighted)	Rao-Scott *χ*^2^	*p-*value
Age (years)			0.70	0.873
0–4	104	6.72		
5–9	201	7.42		
10–14	209	6.48		
15–17	102	6.82		
Gender			40.33	<0.001
Boys	403	8.23		
Girls	213	5.32		
Residence			0.01	0.924
Urban	230	6.99		
Rural	386	6.74		
Education level			11.76	0.019
Not enrolled	51	4.94		
Kindergarten	121	8.86		
Primary school	241	6.55		
Middle school	138	6.91		
High school or above	65	6.71		
Total	616	6.88		

### Logistic regression analyses of factors associated with injury among children

Using the occurrence of injury as the outcome variable, a univariate logistic regression analysis was performed to identify factors associated with childhood injuries. Compared to girls, boys were at a higher risk of injury (OR = 1.60, 95% CI: 1.25–2.05, *p* = 0.003). Children attending kindergarten, primary, or middle school, showed elevated risk compared to non-enrolled children. Day students (those returning home daily) had a significantly higher injury risk compared to non-enrolled children (OR = 1.70, 95% CI: 1.32–2.81, *p* = 0.002). Regarding the primary caregiver, children whose primary caregiver was their father (OR = 1.40, 95% CI: 1.21–1.62, *p* = 0.001) or their grandparents (OR = 1.33, 95% CI: 1.03–1.71, *p* = 0.032) had a higher injury risk compared to those whose primary caregiver was their mother. Age, ethnicity, and the parental education showed no statistically significant association with injury occurrence (*p* > 0.05) (Table 2).

**Table 2:** Univariate analysis of factors associated with injury outcome among children.

Variables	Univariate analysis		Multivariate analysis	
	OR (95%CI)	*p-*value	OR (95%CI)	*p*-value
Age groups (years)				
0–4	1	—	—	—
5–9	1.11 (0.84–1.48)	0.394	—	—
10–14	0.96 (0.61–1.53)	0.852	—	—
15–17	1.02 (0.74–1.40)	0.908	—	—
Gender				
Girls	1	—	1	
Boys	1.60 (1.25–2.05)	0.003	1.58 (1.27–1.97)	<0.001
Ethnicity				
Han	1	—	—	—
Hui and others	0.48 (0.03–8.85)	0.571	—	—
Education level				
Not enrolled	1	—	—	—
Kindergarten	1.87 (1.30–2.69)	0.005	—	—
Primary school	1.35 (1.21–1.51)	<0.001	—	—
Middle school	1.43 (1.00–2.04)	0.049	—	—
High school or above	1.38 (0.93–2.05)	0.092	—	—
Boarding status				
Not enrolled	1	—	1	—
Boarding at school	1.19 (0.79–1.79)	0.362	1.19 (0.78–1.82)	0.418
Day student (returning home daily)	1.70 (1.32–2.18)	0.002	1.69 (1.16–2.46)	0.007
Father’s education				
Illiterate/semi-literate	1	—	—	—
Primary school	2.09 (0.65–6.68)	0.177	—	—
Middle school	1.53 (0.62–3.77)	0.305	—	—
High school	2.21 (0.83–5.85)	0.096	—	—
College or above	2.40 (0.84–6.84)	0.090	—	—
Unknown	1.25 (0.71–2.20)	0.382	—	—
Mother’s education				
Illiterate/semi-literate	1	—	—	—
Primary school	1.03 (0.51–2.09)	0.923	—	—
Middle school	0.97 (0.64–1.47)	0.858	—	—
High school	1.44 (0.65–3.21)	0.317	—	—
College or above	1.45 (0.55–3.83)	0.396	—	—
Unknown	1.15 (0.59–2.26)	0.632	—	—
Primary caregiver				
Mother	1		1	—
Father	1.40 (1.21–1.62)	0.001	1.36 (0.91–2.03)	0.140
Grandparents	1.33 (1.03–1.71)	0.032	1.33 (1.06–1.67)	0.013
Other relatives	0.94 (0.11–7.98)	0.944	0.94 (0.25–3.56)	0.932
Others	0.34 (0.08–1.50)	0.129	0.35 (0.08–1.60)	0.174

Variables identified as significant in the univariate analysis (gender, education level, boarding status, primary caregiver) were included in a multivariate logistic regression model. The results showed that boys remained a high-risk group for injury compared to girls (OR = 1.58, 95% CI: 1.27–1.97, *p* < 0.001). Day students (returning home daily) continued to have a higher injury risk compared to non-enrolled children (OR = 1.69, 95% CI: 1.16–2.46, *p* = 0.007). Children whose primary caregiver was their grandparents had a higher injury risk compared to those cared for primarily by their mother (OR = 1.33, 95% CI: 1.06–1.67, *p* = 0.013) (Table 2).

### Distribution of major injury causes by gender and age groups

Overall, falls (3.86%), animal-related injuries (1.63%), and road traffic injuries (0.74%) were the top three causes of injuries among children aged 0–17 years in Henan Province, accounting for 86.92% of all injuries, with falls alone contributing 53.90%. This ranking was consistent across genders.

By age group, falls, animal-related injuries, and road traffic injuries remained the top three causes for children aged 5–9, 10–14, and 15–17 years, while in the 0–4 years group, burns/scalds (0.47%) replaced road traffic injuries as the third leading cause. Falls were the predominant cause across all age groups, with the highest proportion in children aged 0–4 years (63.82%) and the lowest in those aged 15–17 years (47.56%). Animal-related injuries peaked in the 5–9 years group (29.88%), road traffic injuries in the 10–14 years group (16.14%), and blunt force or sharp object injuries were most common among adolescents aged 15–17 years (Table 3).

**Table 3:** Proportion and event rate of different injury causes for childhood by gender and age groups (%, weighted).

Type of injury	Overall		Boys		Girls		0–4 Years		5–9 Years		10–14 Years		15–17 Years	
	Proportion	Injury event rate	Proportion	Injury event rate	Proportion	Injury event rate	Proportion	Injury event rate	Proportion	Injury event rate	Proportion	Injury event rate	Proportion	Injury event rate
Falls	53.90	3.86	55.39	4.78	51.18	2.80	63.82	4.46	51.73	4.08	52.05	3.47	47.56	3.30
Animal-related injuries	22.72	1.63	21.35	1.84	25.21	1.38	17.92	1.25	29.88	2.36	19.53	1.30	19.81	1.38
Road traffic injuries	10.30	0.74	8.65	0.75	13.28	0.73	4.15	0.29	7.18	0.57	16.14	1.08	15.27	1.06
Blunt force injuries	4.84	0.35	6.30	0.54	2.20	0.12	3.57	0.25	5.32	0.42	3.49	0.23	8.26	0.57
Burns/scalds	3.52	0.25	3.48	0.30	3.59	0.20	6.76	0.47	3.90	0.31	2.33	0.16	0.12	0.01
Sharp object injuries	2.67	0.19	3.53	0.30	1.11	0.06	2.23	0.16	1.81	0.14	2.94	0.20	4.77	0.33
Other injuries	2.05	0.15	1.30	0.11	3.43	0.19	1.55	0.11	0.18	0.01	3.52	0.23	4.21	0.29
Total	100	7.16	100	8.62	100	5.48	100	6.99	100	7.89	100	6.67	100	6.95

### Characteristics of childhood injuries by gender

The most common location for childhood injuries was at home (41.73%), followed by school/kindergarten/childcare institutions (19.59%), roads/streets (18.96%), public places (11.34%), public residential areas (4.47%), and other locations (3.91%). The distribution of injury locations differed significantly between boys and girls (*χ*^2^ = 20.96, *p* < 0.001). Leisure activities were the predominant associated circumstance (59.30%), with significant gender differences (*χ*^2^ = 52.46, *p* < 0.001) (Table 4).

**Table 4:** Circumstances and clinical characteristics of childhood injuries by gender.

Groups	Overall^*^	Boys^*^	Girls^*^	Rao-Scott *χ*^2^	*p-*value
Circumstances of injury occurrence					
Location of injury				20.96	<0.001
Home	812,469 (41.73)	516,337 (41.13)	296,133 (42.80)		
School/kindergarten/childcare institution	381,401 (19.59)	285,618 (22.76)	95,783 (13.84)		
Road/street	369,122 (18.96)	200,608 (15.98)	168,513 (24.36)		
Public place	220,805 (11.34)	122,934 (9.79)	97,871 (14.15)		
Public residential area	87,109 (4.47)	66,000 (5.26)	21,110 (3.05)		
Other	76,171 (3.91)	63,747 (5.08)	12,424 (1.80)		
Activity at time of injury				52.46	<0.001
Leisure activity	1,154,656 (59.30)	718,318 (57.22)	436,338 (63.07)		
Sports activity	225,290 (11.57)	186,101 (14.83)	39,189 (5.67)		
Riding/driving vehicle	163,819 (8.41)	71,868 (5.73)	91,951 (13.29)		
Walking	146,794 (7.54)	111,214 (8.86)	35,580 (5.14)		
Studying	130,965 (6.73)	91,044 (7.25)	39,921 (5.77)		
Essential daily activities (e.g., eating, sleeping)	46,725 (2.40)	26,945 (2.15)	19,779 (2.86)		
Housework	28,850 (1.48)	21,916 (1.75)	6,934 (1.00)		
Other	49,979 (2.57)	27,837 (2.21)	22,141 (3.20)		
Clinical characteristics of injuries					
Nature of injury				19.47	0.007
Contusion/abrasion	729,099 (37.47)	434,627 (34.62)	294,472 (42.63)		
Siting/bite/scratch	429,587 (22.08)	262,638 (20.92)	166,949 (24.17)		
Sprain/strain	220,383 (11.32)	144,258 (11.49)	76,126 (11.02)		
Sharp/open wound	205,525 (10.56)	172,609 (13.75)	32,915 (4.77)		
Fracture	190,296 (9.78)	138,349 (11.02)	51,947 (7.52)		
Burn/scald	68,531 (3.52)	43,718 (3.48)	24,813 (3.59)		
Joint dislocation	25,265 (1.30)	6,927 (0.55)	18,338 (2.65)		
Other	77,318 (3.97)	52,119 (4.15)	25,199 (3.65)		
Site of injury				17.23	0.009
Lower extremities	733,724 (37.70)	460,256 (36.67)	273,468 (39.59)		
Upper extremities	594,298 (30.54)	408,680 (32.56)	185,617 (26.87)		
Head	410,615 (21.10)	289,439 (23.06)	121,177 (17.54)		
Trunk	126,388 (6.49)	48,385 (3.85)	78,002 (11.29)		
Multiple sites	36,835 (1.89)	22,954 (1.83)	13,880 (2.01)		
Generalized	12,801 (0.66)	10,319 (0.82)	2,482 (0.36)		
Other	31,342 (1.61)	15,210 (1.21)	16,133 (2.34)		

^*^The data in the table were weighted; weighted numbers (proportions) are presented.

Regarding clinical features, contusion/abrasion was the most common injury type (37.47%), followed by sting/bite/scratch (22.08%), sprain/strain (11.32%), sharp/open wound (10.56%), fracture (9.78%), burn/scald (3.52%), and joint dislocation (1.30%). The distribution of injury nature differed significantly between boys and girls (*χ*^2^ = 19.47, *p* = 0.007). The lower extremities were the most frequently injured body site (37.70%), followed by the upper extremities (30.54%), head (21.10%), trunk (6.49%), multiple sites (1.89%), and generalized injury (0.66%). Although the ranking of top three sites was identical for both genders, the overall distribution between boys and girls was statistically significant (*χ*^2^ = 17.23, *p* = 0.009) (Table 4).

Among injured children aged 0–17 years, unintentional injuries accounted for 98.96% of cases, followed by self-harm/suicide (0.22%), intentional injuries (0.37%), and injuries of unknown intent (0.45%) (data not shown in the table).

### Supervision status at the time of childhood injury occurrence

Among children aged 0–17 years who were injured, a caregiver was present in 54.17% of cases, while no caregiver was present in 44.78%. When a caregiver was present, adults accounted for 97.56% of supervisors, and minors accounted for 1.73%. Notably, in 67.71% of cases where a caregiver was present, the caregiver was not closely monitoring the child’s activity; only 28.86% of caregivers were both present and attentively supervising the child at the time of injury (Table 5).

**Table 5:** Supervision status at the time of childhood injury occurrence.

Categories	Proportion (%, weighted)
Was a caregiver present at the time of injury?	
Yes	54.17
No	44.78
Other	1.05
Type of caregiver	
Adult	97.56
Minor	1.73
Unknown	0.70
Caregiver’s level of attention	
Present and closely supervising	28.86
Present but not attentive	67.71
Other	0.50
Unknown	2.93

### Hospitalization and days off due to injuries

Among children aged 0–17 years who sustained injuries, 82.60% received outpatient or emergency treatment, while 10.71% rested without seeking medical care due to minor injuries. The proportion hospitalized was 6.61, and 0.08% could not access medical care due to reasons such as the distance to healthcare facilities or financial constraints. The average length of hospital stay for childhood injuries was 18 days, and the average number of days off (rest) due to injury was 9 days, with variations by gender and age group.

**Figure 1:**
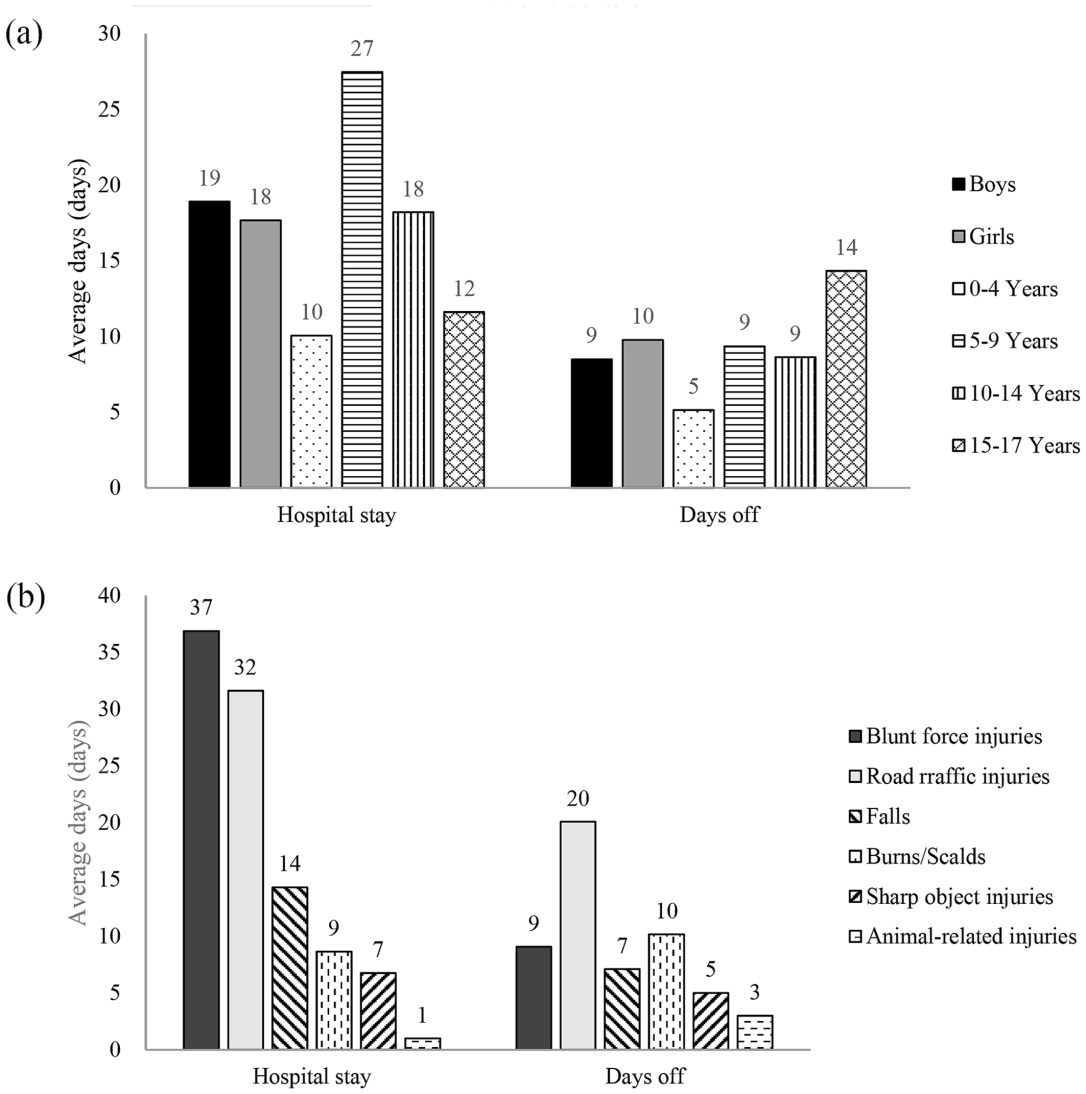
Average hospital stay and days off due to injury by demographic group **(a)** and injury type **(b)**. Data are presented as weighted mean days. “Hospital stay” refers to the average length of inpatient care following an injury; “days off” indicates the mean number of days of restricted activity or absence from regular routines due to injury. Values are stratified by sex, age group, and major injury type. All estimates were adjusted for the complex sampling design.

Regarding specific injury types, blunt force injuries and road traffic injuries had the longest average hospital stays, exceeding 30 days (37 and 32 days, respectively). The average hospital stay for falls was 14 days, while for burns/scalds and cuts/sharp object injuries it was 9 and 7 days, respectively. Road traffic injuries also resulted in the highest average number of days off (20 days). This was followed by burns/scalds (10 days), blunt force injuries (9 days), falls (7 days), cuts/sharp object injuries (5 days), and animal-related injuries (3 days) (Figure 1).

### Economic loss due to childhood injuries

The average cost per injury case among children aged 0–17 years was 2,713.13 Chinese Yuan (CNY). The average cost for boys (3,087.20 CNY) was higher than that for girls (2,027.81 CNY). Across different age groups, children aged 10–14 years incurred the highest average cost per case (4,162.94 CNY), followed by the 15–17 age group (2,580.40 CNY), the 5–9 age group (2,569.00 CNY), and the 0–4 age group (1,418.87 CNY).

Among major injury types, road traffic injuries resulted in the highest average cost per case (8,717.74 CNY), followed by blunt force injuries (6,902.45 CNY), burns/scalds (2,929.51 CNY), animal-related injuries (1,598.58 CNY), and falls (1,498.56 CNY). Other details regarding economic losses are presented in Table 6.

**Table 6:** Economic loss due to injuries among children aged 0–17 years, by gender, age group, and major injury type.

Groups	Economic loss per case (CNY)	Out-of pocket cost per case (CNY)	Reimbursed amount per case (CNY)	Other patient-incurred costed per case (CNY)^*^	Other family-incurred costed per case (CNY)^#^
Overall	2713.13	1817.02	188.03	261.48	450.44
Gender					
Boys	3087.20	2159.59	225.04	275.02	451.11
Girls	2027.81	1189.39	122.07	236.82	449.22
Age groups (years)					
0–4	1418.87	824.54	101.95	94.94	380.70
5–9	2569.00	1787.75	192.69	239.53	436.56
10–14	4162.94	2816.32	300.47	382.40	581.44
15–17	2580.40	1728.30	109.33	357.40	343.46
Major injury type					
Falls	1498.56	861.94	247.58	204.18	286.42
Animal-related injury	1598.58	1010.62	166.71	138.37	231.37
Road-traffic injury	8717.74	6543.59	60.41	609.82	1353.18
Blunt force injury	6902.45	5121.14	120.91	568.79	918.34
Burns/scalds	2929.51	1624.01	97.45	104.74	894.27
Cuts/sharp object injury	1121.98	714.36	87.13	170.95	177.64

^*,#^: “Other Patient-incurred Costs” and “Other Family-incurred Costs” include expenses such as transportation, accommodation, food, nutrition, nursing care, and other property losses incurred by the patient or their family members during the treatment period for the injury.

## Discussion

This population-based cross-sectional study during 2022–2023 suggested that the injury incidence rate among children aged 0–17 years in Henan province of China was 6.88%, and the injury event rate was 7.16%. Using the same injury diagnostic criteria and age-group classifications, our results are higher than those reported in previous surveys conducted in six other provinces in China (4.83%) (22). Henan has long been a major province for labor export in China, resulting in a substantial population of left-behind children and migrant children due to the mobility of their parents. Previous researches indicated that left-behind children have a significantly higher injury incidence rate compared to non-left-behind children (23, 24), which may be an important contributing factor to the higher overall childhood injury rate observed in Henan Province relative to other regions.

The injury incidence rate was significantly higher in boys than that in girls, with boys being at a high-risk group for injury compared to girls (OR = 1.58, 95% CI: 1.27–1.97, *p* < 0.001); this finding was consistent with previous studies (25, 26). The higher injury incidence observed in boys compared to girls may be associated with gender-based differences in activity levels and behavioral patterns. Boys generally engage in more active and risk-prone behaviors, which can increase their exposure to hazardous situations and elevate the likelihood of injury (26). Among children at different education levels, kindergarten children had the highest injury incidence rate (8.66%), and univariate logistic regression analysis indicated that compared to children not enrolled in kindergarten/school, those attending kindergarten were at a significantly higher risk of injury (OR = 1.87, 95% CI: 1.30–2.69, *p* = 0.005). This elevated risk may be attributed to the ongoing development of cognitive and motor skills in younger children, which can increase their susceptibility to injuries-such as falls or collisions during play (27).

Day students (those returning home daily) showed a significantly higher injury risk compared to children not enrolled in school (OR = 1.69, 95% CI: 1.16–2.46, *p* = 0.007). This may be related to their daily exposure to multiple environments-transitioning between home, school, and the community-which increases opportunities for injuries, especially during transportation or unstructured time outside supervised settings (28). This finding is supported by a large cross-sectional study of Canadian school-aged children (*n* = 20,076), which reported that regular active transportation to school over longer distances (approximately >1.6 km) was associated with a 52% higher risk of sustaining a commuting-related injury (OR = 1.52, 95% CI: 1.08–2.15), with evidence of a dose–response relationship. Furthermore, school-age children often engage in more peer-influenced and physically active behaviors, which may involve greater risk-taking without adequate adult oversight during commuting or after-school periods (29).

Children whose primary caregiver was their grandparents were at a higher injury risk compared to those primarily cared for by their mother. The elevated risk in grandparental care may be linked to differing supervisory attitudes. Studies from Hong Kong suggested that, unlike parental caregivers, grandparents who hold a fatalistic worldview and perceive domestic injuries as uncontrollable are likely to have a lower intention to engage in injury prevention practices (30, 31). The disparity in injury risk may also be attributed to differences in the quality of on-the-spot supervision. Furthermore, in Henan, a significant proportion of left-behind children are cared for by their grandparents, a group known to have a higher incidence of injuries compared to non-left-behind children (32–34). Our data reveal a crucial gap: although a caregiver was physically present in 54.17% of injury incidents, effective supervision-defined as close, constant, and undivided attention-was absent in 67.71% of those cases. This suggests that mere physical presence is insufficient. Evidence from a community-based cohort study in China indicates that children under exclusive grandmaternal care tend to have lower cognitive and social–emotional development scores (35). This suggests that grandparents may provide less cognitive stimulation and responsive engagement during supervision. Such a less interactive caregiving style might not only affect child development but also fail to effectively teach safety rules, monitor risk-taking behaviors in real-time, and thus prevent injuries, potentially explaining the elevated injury risk we observed.

Falls were identified as the most common type of childhood injury in this study, which consistent with prior researches (36, 37). This further underscores the importance of maintaining a strong focus on fall prevention within broader, comprehensive strategies for injury control. This study identified the home environment as the most common location for childhood injuries (41.73%), with leisure activities being the most frequently associated circumstance (59.30%). The predominant injury type was contusion/abrasion (37.47%), and the lower extremities were the most frequently injured body site (37.70%). This pattern of injury epidemiology is consistent with the findings from surveys conducted in six other provinces in China (22). The predominance of home as the injury location aligns with both domestic and international evidence, which shows the home environment as the primary setting for childhood injuries (38, 39). In addition, our results indicated that injuries are not merely random accidents but often occur during predictable routines of childhood development. Therefore, prevention strategies should aim to make these necessary and beneficial leisure activities safer, rather than merely restrictive. The consistency of this pattern across multiple provinces suggested (39) that it may represent a public health concern not uncommon in similar regional contexts, and the development and implementation of standardized, evidence-based home safety and active supervision programs should be considered.

The prolonged average hospital stay and substantial hospitalization costs quantified in this study underscore the health consequences and economic burden of childhood injuries, which consist with other studies (40, 41). This burden is not evenly distributed; injuries from mechanisms like road traffic crashes consistently incur the highest medical costs in this study. Therefore, preventing childhood injuries is not only crucial for health but also represents a cost-effective strategy from a health economics perspective.

This study has several limitations. First, it focused on obtaining the overall prevalence, influencing factors, and burden of injuries for 0–17 years children in Henan Province. Because the influencing factors differ significantly across different types of injuries, other factors and those influencing the occurrence of specific injury types were not investigated. Second, the proportion of injury-related disabilities and deaths in the survey was small. This is likely because such events involve psychological trauma for patients and their families, making effective detailed questionnaire surveys difficult. Therefore, the assessment of child injury-related mortality and disability was limited. Third, the use of an operational definition for injury events may have led to an underestimation of the true incidence. This could be due to minor injuries not receiving medical treatment, low healthcare-seeking rates among populations in economically disadvantaged or medically underserved areas, and reliance on informal medical care. Fourth, the retrospective survey design may be affected by recall bias, particularly for minor injuries or specific details such as associated costs. Additionally, injuries among older children and adolescents occurring outside the home may not have been fully captured, as such events were not always known to their caregivers.

Despite these limitations, this study provides the first population-based, provincially representative estimates of childhood injury incidence in Henan Province. The identification of high-risk groups (boys, school-aged children, day-time students, those in specific caregiving arrangements) and high-risk contexts (home, leisure time) allows for targeted interventions. Prevention programs should prioritize fall prevention, enhance home safety assessments, and promote active supervision during play. School-based safety education could be tailored differently for boys and girls based on their predominant injury patterns. Future research should move from descriptive epidemiology to intervention studies, testing the effectiveness of specific home-visit safety programs, community-based awareness campaigns, or school curriculum changes in the local context. Longitudinal studies are also needed to understand the long-term consequences of childhood injuries and to evaluate the sustainability of prevention efforts.

In conclusion, this study identified demographic risk factors, characterize the predominant injury profile, and quantify the associated health and economic costs or children 0–17 years in Henan province of China. This evidence underscores the necessity of moving from general awareness to targeted action. Effective prevention strategies should prioritize home safety interventions, promote supervision during unstructured play, and address the specific risks associated with caregiving by fathers and grandparents. Implementing such evidence-based, context-specific measures is essential to reduce the incidence of childhood injuries and mitigate their long-term impact on children’s health and development.

## Data Availability

All relevant data is contained within the article. The original contributions presented in the study are included in the article/supplementary material, further inquiries can be directed to the corresponding author/s.
